# Hemiablative Focal Low Dose Rate Brachytherapy: A Phase II Trial Protocol

**DOI:** 10.2196/resprot.5433

**Published:** 2016-06-13

**Authors:** Ana Fernandez Ots, Joseph Bucci, Yaw Sinn Chin, David Malouf, Andrew Howie, Komiti Ese Enari

**Affiliations:** ^1^ Cancer Care Centre Radiation Oncology St George Hospital Sydney Australia; ^2^ St George Hospital Urology Sydney Australia

**Keywords:** cancer research, prostate, focal therapy

## Abstract

**Background:**

The objective of focal brachytherapy (BT) is to provide effective prostate cancer control for low-risk disease but with reduced genitourinary, gastrointestinal and sexual side effects in a cost-effective way.

**Objective:**

The aim of this study is to describe a phase II study examining technical and dosimetric feasibility and toxicity, quality of life changes, and local control with post-treatment biopsy outcomes in men with early stage low volume prostate cancer treated with focal iodine-125 seed BT.

**Methods:**

The study design is a prospective, multicenter trial with a planned sample size of 20 patients including men with a minimum age of 60 years, a life expectancy estimated to be greater than 10 years, with low or low-tier intermediate risk prostate cancer, unilateral disease on the biopsy, and a Gleason score of ≤3+4 and <25% cores involved. The investigations specific for the study are multi-parametric magnetic resonance imaging (Mp-MRI) baseline, at 20 and 36 months to rule out high grade disease and a transperineal mapping biopsy (baseline and at 36 months) for more accurate patient selection. The hemigland region will receive 144 Gy. Standard normal tissue constraints will be considered as for a whole gland (WG) implant. Dosimetric parameters will be evaluated at day 30 after the implant. Toxicity and quality of life will be evaluated with international validated questionnaires focusing on urinary, rectal, sexual domain, and general health-related quality of life. The patients will complete this assessment at baseline and then approximately every 6 months after the implant up to 10 years.

**Results:**

To date, one patient is involved in the trial. He underwent the pre-implant investigations which found bilateral disease. Therefore, a standard seed implant was performed. If the results from this trial provide evidence that the treatment is safe, feasible, and improves toxicity, funding will be sought to conduct a large, multicenter, randomized controlled trial (RCT).

**Conclusions:**

This protocol is designed to show feasibility in delivering hemigland focal therapy with seed BT. It may answer crucial questions and obtain data which will enable downstream decisions on focal low dose rate (LDR) prostate BT.

**ClinicalTrial:**

Clinicaltrial.gov NCT02643511; https://www.clinicaltrials.gov/ct2/show/NCT02643511 (Archived by Webcite at http://www.webcitation.org/6ghLCzIhY)

## Introduction

Prostate cancer is the most commonly diagnosed non-cutaneous neoplasm in males in the United States and the second leading cause of cancer mortality. Estimated new cases and deaths from prostate cancer in 2014 were 233,000 and 29,480, respectively. [1].

Widespread screening with the prostate-specific antigen (PSA) test, which can identify patients with asymptomatic tumors that have little or no lethal potential, has decreased the age of diagnosis [2]. With an enormous reservoir of cancers in ageing men, there is a major risk of detection of many cancers that will often never cause symptoms or death [3,4]. Surgery and radiotherapy are widely-employed definitive treatment options; however, their corresponding side-effects include urinary, sexual (ie, erectile dysfunction), and gastrointestinal complications [5]. There are reports showing similar or better outcomes for brachytherapy (BT) versus surgery regarding quality of life in the urinary, bowel or sexual domain [6,7] .

It is increasingly apparent that some older men with low volume favorable cancers and significant medical co-morbidities may be appropriately managed with observation [8]. Recently, active surveillance (AS) has been advocated for selected low-risk cancers with the recognition that Gleason 6 disease is rarely a cause of cancer mortality. A recent cohort study published in 2010 showed, at a median follow-up of 6.8 years, the 10-year prostate cancer actuarial survival was 97.2% in patients undergoing AS [9,10]. After 2 to 3 years of follow-up, approximately one third of patients ceased AS and switched to active therapy [11]. The most common reason for this change was a change in risk classification as a result of repeated biopsies, leading to definitive therapy [12]. Despite the fact that AS was associated with the greatest quality-adjusted life expectancy when compared with definitive treatment of low-risk prostate cancer [13], a considerable majority of men in the United States and Europe who are diagnosed with screen-detected localized tumors still receive aggressive treatment [14,15].

### What is Focal Therapy?

There is increasing evidence that the largest tumor focus within the prostate (called the index lesion) drives the natural history of prostate cancer [16]. Liu and colleagues have shown that metastases in prostate cancer have a common origin (ie, these metastatic cells originate from the same clone) [17]. If the single lesion harboring this metastatic clone could be accurately identified and then conformally targeted, it seems likely that the side effects of treatment for prostate cancer could be reduced due to a smaller treatment volume. Other lesions could then potentially undergo a surveillance approach. The pathological characteristics of the index lesion, namely, the grade and the presence or absence of extracapsular extension, generally indicate the prognosis. Both the index lesion hypothesis and the monoclonal origin of metastatic prostate cancer open the way to a consideration of focal therapy in the majority of men who have multifocal, bilateral disease in which only the clinically important lesion might be ablated [18].

There is no standard definition for focal therapy, but in general it refers to a tissue preservation technique that does not treat the entire prostate gland but instead focuses the treatment to either an index lesion or some defined part of the prostate [19]. This approach is based on the premise that, in appropriately selected men, treating only part of the prostate can be as clinically effective as treating the whole prostate with far less morbidity. This, therefore, is an attractive option for patients where quality of life issues are important. Prostate focal treatment is a potential compromise between definitive treatment and AS. Advances in ultrasound and magnetic resonance imaging (MRI) techniques, as well as tissue sampling, have enhanced the ability to select these patients [20-23].

### Why Low Dose Rate Brachytherapy for Focal Therapy?

A variety of treatment modalities have been used to deliver prostate cancer focal therapy. The literature mainly includes reports on high intensity focused ultrasound (HIFU) [24,25] and cryotherapy [26-28], but neither modality is first line for whole gland (WG) treatment because of toxicity and lack of comparable efficacy.

Low dose rate (LDR) monotherapy has established long-term track record in terms of biochemical control for low and low tier intermediate risk prostate cancer. For favorable risk prostate cancer, LDR BT as monotherapy provides a highly effective treatment option, commonly achieving an undetectable PSA within 4 to 7 years [29,30]. A 15-year biochemical relapse-free survival has been reported at 85.9% for low risk patients [31], and a 10-year disease-specific survival at 96% [32].

The hallmark of LDR prostate BT toxicity is urinary side effects. About 50% will have moderate irritative and obstructive urinary symptoms after the procedure lasting several months. By 12 months, the urinary symptoms of most patients (90%) will return to baseline, although full recovery can be prolonged in 10% to 20% of individuals in 2 to 3 years [33-35]. Mild self-limiting rectal irritation affects 20% to 30% of patients in the first 1 to 2 years after the implant, and rectal bleeding is reported in 2% to 7% of patients [36,37]. The risk of rectal complications has consistently been linked to greater rectal wall doses [38-40]. In a series by Tran et al in which 503 patients were treated with permanent interstitial prostate BT using iodine-125 or palladium-103, 44 patients developed persistent rectal bleeding, including 2 patients with fistula formation. In both of these cases, the volume of rectum receiving greater than or equal to the prescription dose (RV100) exceeded 1 cc [41]. Merrick et al [42] reported on the incidence of BT-related bulbomembranous urethral strictures in a series of 1186 men. The authors described 29 cases (3.6%) noting an association with urethral radiation dose and external beam radiation therapy (EBRT). Kamrava et al [43] compared hemigland BT treatment plans to WG. Hemigland plans revealed a statistically significant decreased radiation dose to organs at risk. The degree of reduction in the dose of 2 cc to these organs was from 64% to 53% for the rectum, 67.5% to 56% to the bladder, and from 95% to 69% to the urethra. One Swiss group used high dynamic range imaging (HDRI) as a partial boost after 64 to 64.4 Gy of EBRT to the prostate, followed by either bilateral or unilateral HDR BT boost. They found no differences in late rectal toxicity and in severe grade ≥3 late urinary toxicity at five years [44]. However, with a background of a significant dose to the WG it is hard to detect a significant decrease in toxicity that this trial aims to achieve.

All curative treatments for prostate cancer have a major potential impact on sexual function. Erectile dysfunction (ED) rates are low at 1 year after the implant with 70% to 80% of men retaining erectile function; this rate declines to around 50% at 5 years post-implant [45]. Although Princess Margaret Hospital published a report on 1111 men with follow-up ranging to over 9 years, with 82% retaining satisfactory erectile function beyond 5 years [29]. Many patients will have improvement in their function with oral phosphodiesterase5 (PDE-5) inhibitors such as sildenafil, vardenafil, and tadalafil [46]. Merrick et al strongly suggested that BT-induced ED is related to the radiation dose delivered to the penile bulb and the proximal crura [47]. As the proximal penis is the most significant treatment-related predictor of BT-related ED, techniques to minimize the radiation dose to the proximal penis such as hemigland may result in improved rates of potency preservation [47].

Apart from the potential of a more favorable toxicity profile due to a dose reduction to the organs at risk with LDR hemigland BT, there are also some technical advantages of delivering LDR BT. LDR is a less invasive procedure that can be done in two stages with a pre-plan before the implant [48] or in a 1-step procedure planned intra-operatively with real-time dosimetry [49]. Although excellent dose distributions can be achieved with pre-planning techniques, intra-operative planning takes into account the intra-operative geometry of the prostate and the surrounding normal tissues. There are methods to dynamically modify the treatment plan as the implant procedure is ongoing based on the coordinates of the deposited seeds such as minimizing the possibility of tumor under dosage and enhancing the conformality of LDR prostate BT [50].

The proposal described here is based on the long-term evidence in the literature that we have for LDR monotherapy for low and low tier intermediate risk prostate cancer over HDR or other techniques. Other treatments available for focal therapy have lack of comparable efficacy and long-term follow-up. It might be more logical to consider whether a therapy might be suitable for focal application after it has been demonstrated to be effective as a WG treatment.

The radiation source used for LDR, iodine-125, has a lower energy of 0.028 MeV giving us more flexibility for planning purposes than the HDR radiation source iridium-192 with 0.38 MeV. Most importantly, prostate seed BT has a post-implant quality verification process where seeds are identified giving us a permanent record of the prostate region treated enabling salvage approaches easier. Focal therapy utilizing LDR BT has been chosen as the above mentioned data provides the most comprehensive treatment modality that addresses the issues of local efficacy, quality assurance verification, excellent toxicity profile, and the ability to localize previous therapy in the setting of potential salvage therapy.

### Summary of the Evidence on Brachytherapy on Focal Therapy

While the use of LDR BT for WG treatment is very well established, there is little data with its use in focal-only treatment. LDR has been delivered to the peripheral zone alone identified by intra-operative magnetic resonance imaging (MRI) [51] with a biochemical free survival (BFS) for low risk patients at 5 and 8 years that was acceptable at 95.6% and 90.0%, respectively, using the Phoenix definition plus PSA velocity greater than 0.75 ng/ml/year. However, the results were poor for intermediate risk patients with a 5 and 8 year progression-free survival (PFS) at 73% and 66%, respectively. Cosset et al [52] have reported their preliminary results on the first 21 patients treated with focal BT with iodine-125 loose seeds targeting the positive region in the biopsy within the prostate and the suspicious sites on MRI, an approach called ultra-focal (UF) BT. After 12 months of follow-up only, a borderline advantage was seen in the International Prostate Symptom Score (IPSS) recovery at 6 months after the implant when compared with a previous cohort treated by WG treatment (*P* =.04). The pre-implant dosimetric parameters for the UF volume with a minimum dose received by the 90% of the prostate volume (D90) of 183.2 Gy and the volume of the prostate receiving 100% prescription dose of 99% (V100) were successfully achieved [52]. Currently, there are three active phase II trials using LDR BT as focal therapy (Table 1). Morris et al are recruiting patients with low or low tier intermediate risk after MRI elastography transrectal ultrasound biopsy targeting high grade areas [53]. In France, Bachaud is recruiting patients with low risk prostate cancer for a focal target seed implantation [54], and Langley et al in the United Kingdom are evaluating side effects, quality of life, and cancer control in patients with prostate cancer diagnosed on only one side of the prostate gland [55]. Zelefsky opened a phase II study for men with early-stage low-risk prostate cancer treated with hemigland and focal LDR BT examining the tolerance profile. Unfortunately, this trial was terminated due to lack of accrual. [56].

The aim of this study is to address the toxicity, feasibility, and utility of hemiablative focal LDR BT as treatment for localized prostate cancer. We hypothesize that this form of LDR BT is safe and will give similar disease control outcomes when compared to established WB treatment techniques, but with decreased toxicity leading to an improved quality of life. As this is a feasibility study, we are looking at toxicity and safety (adequate implant) in highly selected candidates.

### Trial Objectives

The trial objectives for this study are described in Textbox 1.

**Table 1:** Phase II studies using low dose rate focal brachytherapy.

Study details	Phase II study			
	Morris et al [53]	Bachaud [54]	Langley et al [55]	Zelefsky [56]
Location	British Columbia Cancer Agency (BCCA), Canada	Institut Claudius Regaud, France	Royal Surrey county Hospital NHS, United Kingdom	Memorial Sloan Kettering Cancer Center, United States
Current progress	Recruiting	Recruiting	N/A	Terminated in February 2016 due to lack of accrual
Treatment	LDR focal BT	LDR focal BT; prescription dose (PD) 160 Gy +/- 5%	LDR BT hemigland 145 Gy	Hemigland LDR BT; PD 144 Gy
Patients^a^, n	10	17	34	80
Stage	≤T2a	≤T2a	≤T2b	≤T2a
Gleason	≤3 4≤2 cores	≤3+3	≤4+3	Up to Gleason 7 in just 2 cores
PSA	<10	<10	<15	<10
Inclusion tests	Transrectal ultrasound (TRUS)	3D prostate mapping biopsy, MRI	Transperineal template-guided mapping (TTGM) multi-parametric magnetic resonance imaging (Mp-MRI)	TRUS
Primary outcome	To fit for focal disease and adequate treatment plans	Successful post-implant dosimetry	Urinary, sexual and bowel toxicity, and quality of life	Late toxicity
Secondary outcome	Quality of life, treatment evaluation	Progression-free survival (PFS; (Phoenix definition), qualify of life, biopsy, toxicity	Tumor control	Efficacy, quality of life, post-treatment MRI vs post-biopsy
Timeframe, years	4	3	5	2

^a^Open estimate.

**Trial objectives.**

Objectives

- Primary objective
  To demonstrate the feasibility of delivering hemigland focal therapy (the delivery of the prescription dose to the half of the prostate) with a seed BT implant in a multi-center Australian study.
- Secondary objectives
  - To determine acute and late rectal, urinary, and sexual toxicity following hemiablative iodine-125 brachytherapy (BT) treatment.
  - To assess the change from baseline in quality of life indicators at specific time intervals using the following validated international questionnaires after hemiablative iodine-125 (BT) treatment:
    - International Prostate Symptom Score (IPSS)
    - International Index of Erectile Function (IIEF)
    - Expanded Prostate Cancer Index (EPIC)
  - To evaluate the local tumor control in terms of biopsy outcomes after focal BT 36 months after the treatments.
  - To compare target coverage and relative doses to the rectum and the urethra for the same patient performing a hemigland treatment planning versus WG treatment planning, and compare rates of toxicity and quality of life after hemigland implant with historical WG cohorts.

## Methods

### Study Design

This multi-institution, prospective phase II trial aims to determine whether hemiablative treatment with LDR for prostate cancer is dosimetrically safe and feasible. This study will record data for patient quality of life parameters, in particular in terms of urinary, rectal, and sexual function side effects.

### Study Group

Patients with ipsilateral low grade disease (N=20) will be evaluated prospectively after hemiablative focal therapy. Because of the historically small size of trials in this area, probably reflecting the difficulty of recruitment of patients in this scenario, a pilot will be conducted to establish the feasibility of a full scale trial prior to committing to a large study.

### Eligibility Criteria

**Figure 1:**
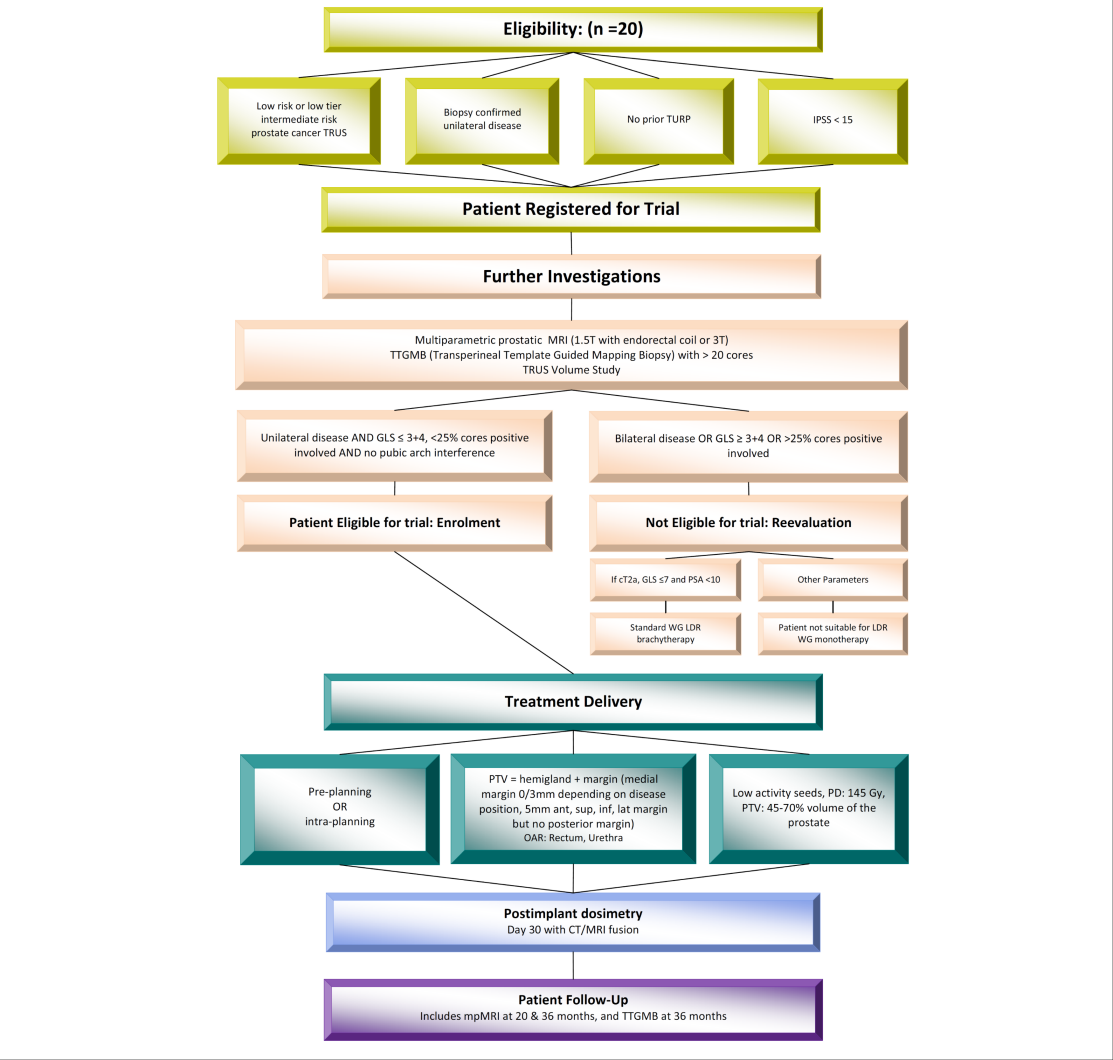
Trial schema.

To be eligible for the study, participants must meet each of the eligibility requirements (Textbox 2). If a participant has one of the following, they will be ineligible for the study: (1) does not meet staging criteria for low risk or low tier intermediate risk prostate cancer, (2) bilateral prostatic disease, (3) prior hormonal therapy, (4) recent IPSS more than 17, (5) unfit for general anesthetic, (6) MRI contraindicated, (7) unable to cease anticoagulant therapy, and (8) life expectancy less than 10 years. The trial schema is shown in Figure 1.

**Eligibility requirements.**

Requirements

Patients must have histologically proven adenocarcinoma of the prostate.

Patients must have low or low tier intermediate prostate cancer.

Low risk prostate cancer patients must have:

- Clinical stage ≤ T2a
- Gleason score of 6 and iPSA ≤10 ng/ml
- Less than 25% cores positive

Low tier intermediate risk patients may have:

- Clinical stageT2a
- Gleason score ≤3+4=7
- PSA ≤10 ng/ml
- Less than 25% cores positive

Patients must be fit for general anesthetic.

Patients must have unilateral disease on biopsy.

Patients must have an Eastern Cooperative Oncology Group (ECOG) performance status of 0-2.

Men ≥60 years of age with a life expectancy estimated to be >10 years.

Patients must have no contraindications to interstitial prostate BT.

International Prostate Symptom Score (IPSS) ≤16

Patients on anticoagulant therapy must be able to stop therapy safely for at least 7 days.

Patients must not have any contraindications to MRI.

### Pre-Treatment Evaluation

**Figure 2:**
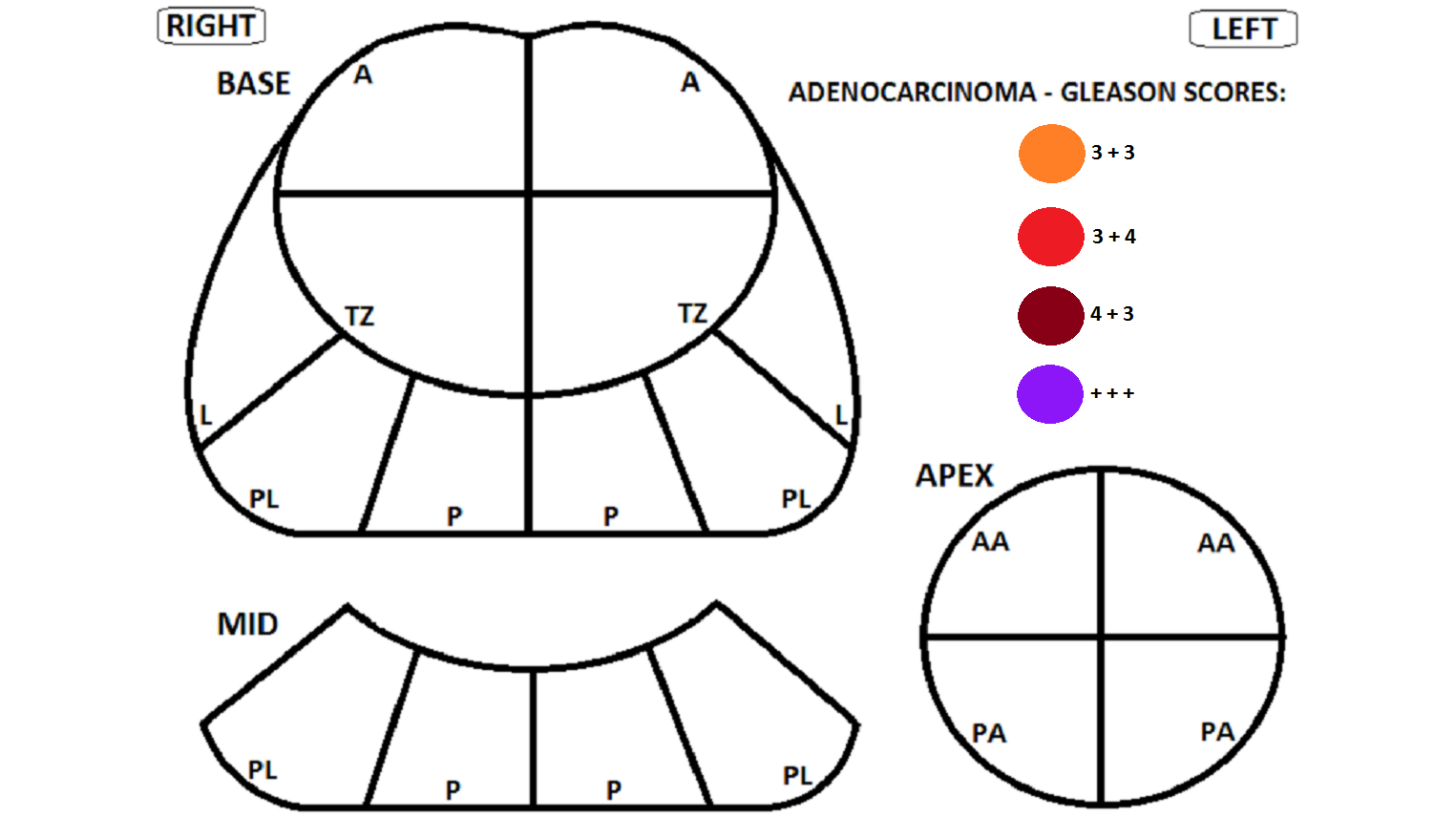
Transperineal template. Diagram prepared by Warick Delprado at Douglass Hanly Moir Pathology and used by permission.

The components of the pre-treatment evaluation are shown in Textbox 3. The transperineal template is diagramed in Figure 2.

The protocol includes a screening phase with multi-parametric magnetic resonance imaging (Mp-MRI) and mapping biopsy for patient selection. Improved imaging techniques coupled with better sampling of the prostate [57,58] allows to identify men with low volume focal disease selection who may be suitable for tissue preservation strategies. It has been estimated that between one half and two thirds of men with prostate cancer may be amenable to some form of focal therapy [59,60].

**Pre-treatment evaluation.**

Pre-treatment

Patient demographics and histological information from transrectal ultrasound (TRUS) prostate biopsy will be collected.

For preplanning technique, ultrasound volume study to assess prostatic volume and pubic arch interference will be performed.

A multi-parametric prostatic MRI (Mp-MRI) (1.5 T with endorectal coil or 3T). The data set should include T1-weighted, T2-weighted, diffusion-weighted, and contrast-enhanced MRI. Imaging could be adequately performed at 1.5 T with endorectal coil or 3T without endorectal coil [57]. Suspicion of bilateral disease or high grade unilateral disease will exclude the patient from the study. Biopsy of any suspicious target lesions as identified on Mp-MRI is recommended.

If a TRUS biopsy has only been performed then the patient will also undergo a transperineal template guided mapping (TTGM) biopsy, with a minimum of 20 cores obtained, at the time of the volume study (for preplanning technique) (Figure 2). A single anatomical pathologist will review the biopsy. In addition the following parameters will be recorded to allow for stratification according to:

- T stage: T1c vs T2a
- Gleason: 3+3=6 vs 3+4=7
- Presence of lymphovascular invasion
- Presence of perineural invasion

Baseline quality of life assessments:

- International Prostate Symptoms Score (IPSS) questionnaire
- International Index Erectile Function (IIEF) questionnaire
- Expanded Prostate Cancer Index Composite (EPIC) questionnaire

### Informed Consent, Registration, and Enrolment

All patients must sign an informed consent form to be registered in this study. Completed consent forms will be sent to the Radiation Oncology Clinical Trials Office at the St George Cancer Care Centre (STGCCC) and eligibility will be confirmed by the study coordinator. A patient identification number (PIN) will be assigned to the patient. The registration process will include the consent and the prostate Mp-MRI and a transperineal template-guided mapping (TTGM) biopsy of the prostate to rule out high grade disease and ensure eligibility for focal therapy. Only patients still suitable for focal therapy after these studies will be enrolled in the trial and will go ahead with the treatment. They will be followed up to 10 years. Patients not suitable for the trial will go ahead with standard WG BT if they still wish to have active radiation treatment. This is a feasibility study to assess the feasibility of this technique with a permanent seed implant to half of the prostate in terms of acute side effects and post-implant dosimetry parameters. Normal tissue tolerance will have the priority in the planning algorithm. There is no randomization process.

### Investigations

All investigations required before study entry are standard for this grade and presentation of prostate cancer and include a TRUS-guided prostate biopsy for diagnosis and a PSA test within the last 3 months. Investigations specific for the study include a Mp-MRI of prostate using either a 3 Tesla magnet alone or a 1.5 Tesla magnet and an endorectal coil. A baseline Mp-MRI should be performed at least 6 to 8 weeks after the initial biopsy and the Mp-MRI should be repeated at 20 and 36 months after treatment or earlier if PSA is detected to be rising during follow-up. Patients should be scanned on the same scanner for all three examinations, especially when tracking changes in apparent diffusion coefficient (ADC) values over time. A central radiology review of the images will take place in Southern Radiology by Dr Jonathan Seef. In addition, a TTGM prostate biopsy with a minimum of 20 cores and at 36 months post treatment will be included. Central pathology review of the specimens will take place in a center assigned in each participating Australian state.

### Interventions

**Figure 3:**
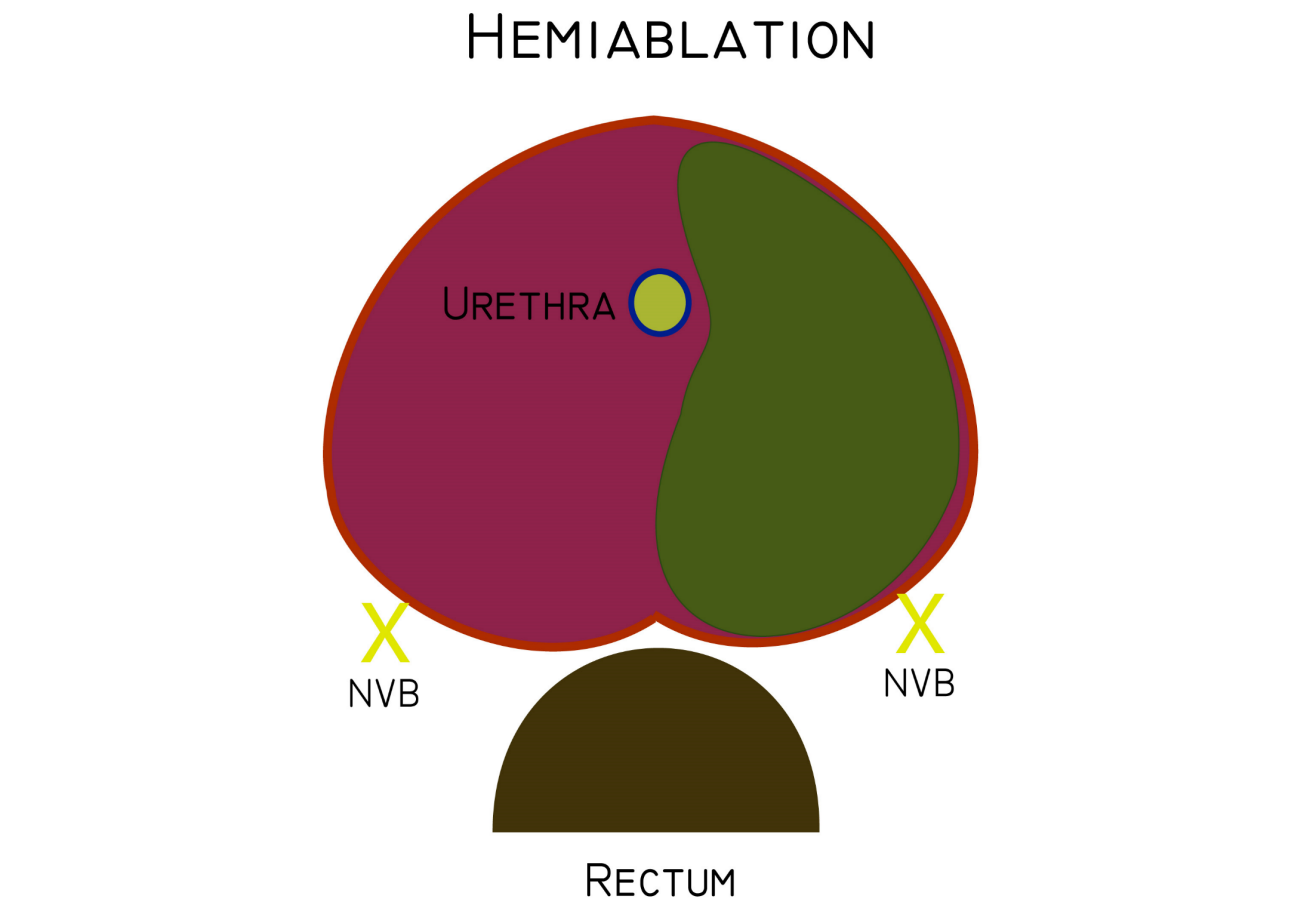
Hemiablation.

A preplanned technique or intra-operative planning technique will be used depending on the individual institutional preference. Patients participating in this study will undergo hemigland LDR prostate BT performed by experienced BT teams that perform a minimum of 40 cases per year (Figure 3).

The prostate volume study will be performed under sedation or general anesthesia, and patients will be set up in the dorso-lithotomy position. The urethra will be identified by insertion of aerated gel allowing visualization on ultrasound. A TRUS volume of the prostate will be performed. A set of 5 mm slice images are acquired using the transverse mode for 3D reconstruction of the prostate. Pubic arch interference will be identified in case modification of probe angle or leg position if necessary.

For the hemigland preplanning, the pre-implant clinical target volume (CTV) will be defined as half of the prostate excluding the urethra, as visualized and contoured on the reconstructed ultrasound images. The planning target volume (PTV) will be created by adding a 3 to 5 mm margin around the CTV except for the posterior margin that will be 0 mm. The PTV will encompass 45% to 70% of the total prostate volume maximum. A hemiablative approach over two different focal therapy scenarios has been chosen, as shown in Figure 1, with hemiablation or zonal ablation (hockey stick 5 mm beyond midline). An experienced medical physicist will plan the iodine-125 seed and needle positions achieving dosimetric parameter goals. All plans will be approved by both the responsible radiation oncologist and a medical physicist, both of whom are experienced in prostate BT; the STGCCC team of investigators has performed more than 1000 prostate BT procedures.

The treatment planning dosimetric parameters used for optimization aim for prostate PTV V100 is greater than 98%, with a V150 of 55% to 65% and V200 of less than 20%. (V100, V150, and V200 are the percentage of the prostate volume covered by the prescription isodose, the 150%, and the 200% isodose, respectively). Urethral dose (UD) is usually described as the UD5 or UD30, which are the dose to 5% and 30% of the urethra, respectively. UD5 is representative of a urethral maximum dose and should be less than 150% of the prescription dose, while UD30 should be less than 125%. For the rectum, the rectal volume in cubic centimeters receiving 100% of the prescribed dose (RV100) is commonly used and should be less than 1.3 cm^3^for day 30 dosimetry.

In addition, a whole prostate LDR planning will be performed for a potential matched historical control toxicity comparison with patients from the existing large LDR database based on standard whole prostate dosimetric features as well as other clinical features like prostate volume, IPSS and potency, and medical conditions (eg, diabetes). This will enable us to report the difference in dose for the two plan sets for the targets hypothesized to be responsible for toxicities like urethritis or proctitis. This will require a pre-implant CTV defined as the prostate excluding the urethra, as visualized and contoured on the reconstructed ultrasound images. The PTV will be created adding a 5 mm margin around the CTV except for the posterior margin that will be 0 mm. The same planning dosimetric parameters will be recorded as for the hemigland treatment planning.

For the implant procedure, the patient will be under general anesthesia and set up in the dorsal lithotomy position on the operating table in the BT theatre as per the local institutional seed implant protocol. A TRUS volume of the prostate will be matched to the images of the preplanned study. An intra-operative approach is also acceptable. Iodine-125 seeds will be inserted under US template and fluoroscopy guidance according to the plan.

At the completion of the needle and seed insertion, the Foley catheter will be kept in place and the patient will be taken to the post-anesthesia recovery (PAR) area. Patients will be discharged from PAR when they have made an appropriate recovery from anesthesia and have successfully voided urine after the Foley catheter is removed.

Patients participating in this study will be followed in the usual fashion and only require the following additional tests beyond those of standard practice: Mp-MRI at 18 and 36 months and TTGMB at 36 months.

### Patient Follow-Up

#### Postimplant Dosimetry

Post-plan quality assurance using magnetic resonance computed tomography (CT) fusion (MR-CT fusion) is required because it reduces inter-observer variation by improving prostate edge detection, and allows appreciation of treatment margins [61,62]. Fusion can be accurately performed to sub-millimeter accuracy using the seeds as fiducial markers. It is essential to use the appropriate MR sequences in order to facilitate both prostate contouring and seed localization. A Fast Spin Echo T2-weighted MR sequence is used, with the technical parameters described in Textbox 4.

**Technical parameters used with the Fast Spin Echo T2-weighted MR sequence.**

Parameters

- Repetition time (TR) = 4500 msec
- Echo time (TE) = 90 msec
- Echo train length (ETL) =10,
- Pixel bandwidth (BW) = 580 Hz/pixel
- Field of view = 20 x 20 cm
- 3 mm slice thickness, 0 mm gap
- Acquired matrix size = 320 x 224 with phase encoding direction along rows
- Flip angle = 90°

CT images are likewise obtained in the supine position, imaging the prostate and all seeds visible on the scout image in 2 mm slices. Aerated gel with Ultravist contrast is inserted for the pelvic CT for urethral localization by the oncologist. No specific bowel preparation is used before either scan but they should be performed sequentially, with the CT following the MRI generally within half an hour. Patients will also undergo a pelvic and chest x-ray to assess for any seed migration.

In order to improve this pilot trial consistency and check the hemi-implant quality, MR-CT fusion for all the participants will be reviewed by one of the two primary investigators with experience in MRI contouring and MRI-CT fusion; Dr A Fernandez has performed more than 100 MRI-CT fusions.

#### Follow-Up Schedule

Routine assessment following completion of treatment includes PSA and clinical evaluation 4 to 6 weeks after the implant to manage and document any acute toxicity. The assessment will continue every 3 to 6 months depending on symptoms up to 3 years, and then every 6 to 12 months, with a PSA and digital rectal exam at each visit beyond 6 months up to 10 years. The IPSS, the International Index of Erectile Function (IIEF), and the Expanded Prostate Cancer Index Composite (EPIC) questionnaire are recorded at baseline and at each visit as is standard for all men receiving any type of prostate BT in the STGCCC. Specific follow-up for the study will involve a repeat Mp-MRI by 20 and 36 months and a TTGMB at 36 months. Patients will be followed up to 10 years after the implant, although the study finishes after 36 months of enrollment. Once we reach a median of 3-year follow-up (ie, sufficient time to determine rate of local control with the biopsies in 50% patients), then it would be adequate to commence the randomized controlled trial (RCT).

### Exit Strategy and Rescue Therapy

Biochemical failure will follow a PSA velocity greater than 0.75 ng/ml years in addition to nadir +2. Both parameters appear to better predict clinical failure after therapies that target less than the WG. Failure may also be proven on the TTGM control biopsies at 36 months or earlier in case of biochemical failure defined by Nguyen et al [51].

Definition of treatment failure will involve relapse in PTV or outside our PTV confirmed by biopsy. Relapse outside the PTV is not essentially a local treatment failure, and may be due to suboptimal patient selection. Due to the additional resources required to continue on this trial, these cases will be recorded as a treatment failure for further validation of the hemifocal BT as a treatment alternative to WG therapy. Interval from treatment to failure will be recorded.

After biochemical or local failure, the patient will exit the study. After appropriate investigations different salvage therapies can be offered to the patients who have failed. For local failure radical prostatectomy, HDR salvage BT or salvage cryotherapy in case of ipsilateral or contralateral relapse, or hemigland focal seed BT in case of contralateral relapse alone will be initiated. A choice of the treatment will be made after appropriate discussion in a multidisciplinary meeting and will be individualized.

### Study Endpoints

#### Primary

Feasibility of the hemigland focal therapy (the delivery of the prescription dose to half of the prostate) using a BT seed implant, while respecting standard tolerance doses of adjacent normal organs is the primary study endpoint.

#### Secondary

Secondary study endpoints are (1) assessment of rectal, urinary, and sexual toxicity following hemiablative prostate seed BT; (2) change from baseline in quality of life indicators at specific time intervals using validated international questionnaires (IPSS, IIEF, EPIC) following hemiablative iodine-125 BT treatment; (3) evaluation of local tumor control in terms of biopsy outcomes after focal BT 36 months after completion of therapy; and (4) comparison of target coverage and relative doses to the rectum and the urethra for the same patient performing an hemigland treatment planning versus WG treatment planning and toxicity and quality of life after hemigland implant comparison with historical whole gland cohorts.

#### Measurement of Endpoints

##### Feasibility

Dose parameters for prostate (V100, V150, V200), rectum (V100), and urethra (D5, D30) will be recorded for the hemigland and the WG treatment planning. Dosimetric parameters in the post-implant dosimetry at day 30 for WG BT are well defined in the literature and they are correlated to efficacy and toxicity. The post-implant dosimetric study for hemigland BT patients will aim for the same dosimetric parameters at day 30 after the implant for tumor coverage and organs at risk dose.

##### Toxicity Evaluation Assessment

**Figure 4:**
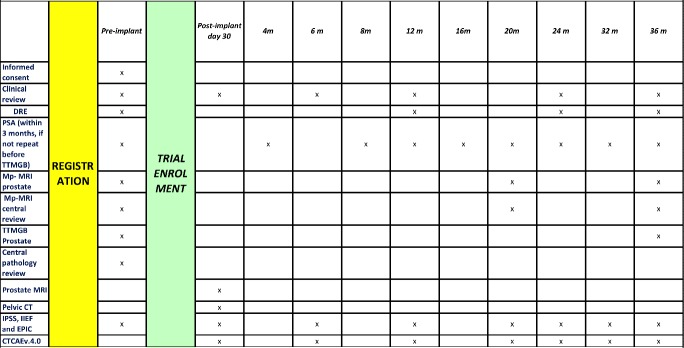
The necessary data to be collected at various time points of the study.

Patients will be assessed on the day of enrollment, at 4 to 6 weeks, and every time they come back for follow-up (every 6 months) to determine acute, sub-acute, and late toxicity after the implant in the urinary, rectal, and sexual domain. An adverse event is any deleterious effect which may occur as a result of the intervention. Treatment-related toxicities will be graded using the National Cancer Institute Common Terminology Criteria for Adverse Events (CTCAE) Version 4.0 as follows: (1) grade 0 is no adverse events, (2) grade 1 is mild events not requiring intervention, (3) grade 2 is moderate events interfering with normal activities, (4) grade 3 is severe events causing inability to carry out normal activities, (5) grade 4 is life threatening or disabling events, and (6) grade 5 is death (Figure 4). A follow-up assessment will be undertaken within seven days in patients reporting greater than grade 1 toxicities, and will continue in this manner until toxicity has resolved, with documentation of action taken at each time point. Failure of an adverse event to resolve and its translation to chronicity at a given time point will be documented by the clinician. Serious adverse events (grade 3 or greater) will require assessment by the clinician and documentation of action taken.

##### Quality of Life

Quality of life will be assessed using the IPSS, IIEF, and EPIC questionnaires. The change from baseline will be measured at specific time points (Multimedia Appendix 1).

##### Local Tumor Control

Local tumor control will include negative or indeterminate TTGMB 36 months after the treatment within the whole prostate. Definition of treatment failure will involve relapse in PTV or outside our PTV and confirmed by biopsy. In addition, dosimetric parameters and toxicity rates will be compared with historical WG treatment cohorts.

### Data Collection

The necessary data to be collected at various time points of the study is outlined in Figure 4.

### Statistical Considerations

It is anticipated that a sample size of 20 patients will be accrued to enable the investigator to deliver hemiablative seed BT with a reasonable amount of confidence. This feasibility study will proceed in 5 participating centers. Failure to accrue 15 patients in 24 months will initiate early closure of this study. The time-frame for completion of recruitment will be approximately 12 months, with a further 24 months required for collection of acute and late toxicity data, maturation of quality of life data, and correlation with the biopsy by 36 months.

We do not consider it necessary to use early stopping rules for poor quality implants (not adequate dosimetric parameters on day 30), or acute treatment-related toxicities because of the low number of patients we are aiming for. An analysis will be performed after 20 patients have participated in the study. Therefore, if greater than 20% of patients have poor quality implants, this procedure will be unacceptable.

Descriptive statistics will be used to describe the frequency at which prostatic cancer low grade disease can be seen on Mp-MRI, and the frequency of unilateral prostatic disease is found after a mapping of the prostate. In addition to standard PSA follow-up, response will be assessed by imaging at 20 and 36 months as well as by TRUS-guided biopsy at 36 months.

Data analysis will be undertaken by Dr Ana Fernandez with the assistance of a qualified statistician.

### Resources and Implementation

#### Ethics

The application for the South Eastern Sydney Local Health District (SESLHD) Human Research Ethics Committee has been recently approved.

#### Skills and Resources

Three of the investigators are experienced BT proceduralists with extensive experience in the use of TRUS and transperineal needle implantation. The fourth investigator is a BT fellow in training and will be assisting with the procedure.

Planning systems will be institution-dependant but must allow appropriate collection of the above mentioned data. Acceptable planning systems will include Variseed, Nucletron First, PSID, and MIM Symphony. TGA-approved iodine-125 seeds may be purchased from any of the vendors within Australia.

#### Data Collection

The necessary data to be collected at various time-points of the study is outlined in Figure 4. The principal and co-investigators will be responsible for collection of this data. This data will be manually entered onto paper forms and transferred to a secure database which is password protected and accessible only to the investigators.

#### Budget and Funding

The investigations beyond normal practice that are required for this protocol are (1) two TTGM biopsies of the prostate, one pretreatment and another one at 36 months following treatment; and (2) Mp-MRI of the prostate, required at baseline and again 20 and 36 months following completion of treatment to assess response.

The post-implant quality assessment will require a a non-Mp prostatic MRI at day 30. This is not our standard practice but we arrange it sometimes for specific patients that require more accurate post-implant quality evaluation. Apart from the 20 month and 36 month assessments, follow-up after completion of treatment is standard. The costs entailed by the MRI examinations and the TTGM prostate biopsy will be billed to the Internal Hospital Research Budget. Internal funding prior to commencement will be undertaken by each institution. SGCCC has already coordinated local funding using trust fund cost centre number and hence can proceed immediately after ethics approval. A grant application will cover part of the expenses. There will be no out-of-pocket cost for the patients enrolled in the study.

## Results

The study opened for recruitment in September 2015. One patient has been involved so far in the study that initially met the eligibility criteria and signed the consent. During the pre-treatment evaluation process he underwent a Mp-MRI which showed Prostate Imaging Recording and Data System Score 5 (PI-RADS 5) on the left lobe and PI-RADS 4 on the right lobe. A PI-RADS 5 represents clinically significant, highly likely to be present and PI-RADS 4 is clinically significant and cancer likely to be present [63]. Subsequently, as per protocol, a TTGMB was performed finding a Gleason score of 3+4= 7 in 3 cores on the right side and in 8 cores of the left side. The histopathology report was reviewed. The patient underwent a standard seed implant prescribing 144 Gy to the whole prostate.

## Discussion

This protocol is designed to show feasibility in delivering hemigland focal therapy with seed BT. Our whole-prostate seed BT program has been running for more than 10 years with excellent results (data not published). We believe that our well-established experience with seed BT will ease the performance of the hemigland technique. The reduction in toxicity can be of significant importance, particularly in a well-selected population, therefore we consider the pre-implant investigations crucial.

If the trial is successful, showing feasibility meeting the dosimetric parameters in the post-implant setting, toxicity parameters, quality of life, and tumor local control will be evaluated. Once evaluated we will be able to move towards a RCT scenario comparing standard WG seed implant versus hemigland to confirm the benefit in toxicity and quality of life with the highest level of evidence. Cost effective analysis should be performed in the future given the cost of the pre-implant investigations before establishing the focal therapy technique.

Furthermore, this protocol will give us opportunities to study more data, such as the correlation between Mp-MRI findings with TTGMB which is still in early stages [64,65].

## Appendix

Multimedia Appendix 1 Quality of life instruments: Expanded Prostate Cancer Index Composite (EPIC) questionnaire, International Prostate Symptoms Score (IPSS) questionnaire, International Index Erectile Function (IIEF) questionnaire.

## Abbreviations

AS: active surveillance
BT: brachytherapy
CT: computed tomography
CTV: clinical target volume
ED: erectile dysfunction
HDRI: high dynamic range imaging
IIEF: International Index of Erectile Function
IPSS: International Prostate Symptom Score
LDR: low dose rate
Mp-MRI: multi-parametric-magnetic resonance imaging
MR-CT fusion: magnetic resonance imaging computed tomography fusion
MRI: magnetic resonance imaging
PAR: post-anesthesia recovery
PI-RADS 4: Prostate Imaging Recording and Data System Score 4
PI-RADS 5: Prostate Imaging Recording and Data System Score 5
PSA: prostate-specific antigen
PTV: planning target volume
RCT: randomized controlled trial
STGCCC: St George Cancer Care Centre
TRUS: transrectal ultrasound
TTGM: transperineal template-guided mapping
UF: ultra-focal
WG: whole gland
