# Supplementary material for: Hemiablative Focal Low Dose Rate Brachytherapy: A Phase II Trial Protocol
Source: JMIR Res Protoc. 2016 Jun 13;5(2):e98. doi: 10.2196/resprot.5433 (PMC4923592; doi:10.2196/resprot.5433)
Supplement: Multimedia Appendix 1 [file resprot_v5i2e98_app1.pdf]

Patient initials: ☐☐☐ Trial Number (*if applicable*): ☐☐☐

Date of Birth: ☐☐☐☐☐☐ Mrn: ☐☐☐☐☐☐☐☐☐

PROSTATE CANCER INSTITUTE

DIVISION OF CANCER SERVICES
ST GEORGE HOSPITAL

EORTC QUALITY OF LIFE QUESTIONNAIRE (QLQ- C30 & PR25)

Today's Date: ____/____/_____

We are interested in some things about you and your health. Please answer all the questions yourself by circling the number that best applies to you. There are no "right" or "wrong" answers. The information that you provide will remain strictly confidential.

		**Not at all**	**A little**	**Quite a bit**	**Very Much**
1.	Did you have any trouble doing strenuous activities like carrying a heavy shopping bag or suitcase?	**1**	**2**	**3**	**4**
2.	Did you have any trouble taking a **long** walk?	**1**	**2**	**3**	**4**
3.	Did you have any trouble taking a **short** walk outside of the house?	**1**	**2**	**3**	**4**
4.	Did you need to stay in a bed or chair during the day?	**1**	**2**	**3**	**4**
5.	Did you need help with eating, dressing, washing yourself or using the toilet?	**1**	**2**	**3**	**4**
	**During the past week:**				
6.	Were you limited in doing either your work or other daily activities?	**1**	**2**	**3**	**4**
7.	Were you limited in pursuing your hobbies or other leisure time activities?	**1**	**2**	**3**	**4**
8.	Were you short of breath?	**1**	**2**	**3**	**4**
9.	Have you had pain?	**1**	**2**	**3**	**4**
10.	Did you need to rest?	**1**	**2**	**3**	**4**
11.	Have you had trouble sleeping?	**1**	**2**	**3**	**4**

		Not at all	A little	Quite a bit	Very Much
12.	Have you felt weak?	**1**	**2**	**3**	**4**
13.	Have you lacked appetite?	**1**	**2**	**3**	**4**
14.	Have you felt nauseated?	**1**	**2**	**3**	**4**
15.	Have you vomited?	**1**	**2**	**3**	**4**
16.	Have you been constipated?	**1**	**2**	**3**	**4**
17.	Have you had diarrhoea?	**1**	**2**	**3**	**4**
18.	Were you tired?	**1**	**2**	**3**	**4**
19.	Did pain interfere with your daily activities?	**1**	**2**	**3**	**4**
20.	Have you had difficulty in concentrating on things like reading or watching television?	**1**	**2**	**3**	**4**
21.	Did you feel tense?	**1**	**2**	**3**	**4**
22.	Did you worry?	**1**	**2**	**3**	**4**
23.	Did you feel irritable?	**1**	**2**	**3**	**4**
24.	Did you feel depressed?	**1**	**2**	**3**	**4**
25.	Have you had difficulty remembering things?	**1**	**2**	**3**	**4**
26.	Has your physical condition or medical treatment interfered with your **family** life?	**1**	**2**	**3**	**4**
27.	Has your physical condition or medical treatment interfered with your **social** life?	**1**	**2**	**3**	**4**
28.	Has your physical condition or medical treatment caused you financial difficulties?	**1**	**2**	**3**	**4**

For the following questions please circle the number between 1 and 7 that best applies to you:

29. How would you rate your overall **health** during the past 2 weeks?

**1**	**2**	**3**	**4**	**5**	**6**	**7**
very poor						excellent

30. How would you rate your overall **quality of life** during the past 2 weeks?

**1**	**2**	**3**	**4**	**5**	**6**	**7**
very poor						excellent

EORTC QLQ - PR25

Patients sometimes report that they have the following symptoms or problems. Please indicate the extent to which you have experienced these symptoms or problems during the past week. Please answer by circling the number that best applies to you.

During the past week:

	Not at all	A little	Quite a bit	Very much
31. Have you had to urinate frequently **during the day**?	**1**	**2**	**3**	**4**
32. Have you had to urinate frequently **at night**?	**1**	**2**	**3**	**4**
33. When you felt the urge to pass urine, did you have to hurry to get to the toilet?	**1**	**2**	**3**	**4**
34. Was it difficult for you to get enough sleep, because you needed to get up frequently at night to urinate?	**1**	**2**	**3**	**4**
35. Have you had difficulty going out of the house because you needed to be close to a toilet?	**1**	**2**	**3**	**4**
36.Have you had any unintentional release (leakage) of urine?	**1**	**2**	**3**	**4**
37.Didyouhavepainwhenyouurinated?	**1**	**2**	**3**	**4**
38. Answer this question only if you wear an incontinence aid. Has wearing an incontinence aid been a problem for you?	**1**	**2**	**3**	**4**
39.Have your daily activities been limited by your urinary problems?	**1**	**2**	**3**	**4**
40.Have your daily activities been limited by your bowel problems?	**1**	**2**	**3**	**4**
41.Have you had any unintentional release (leakage) of stools?	**1**	**2**	**3**	**4**
42.Have you had blood in your stools?	**1**	**2**	**3**	**4**
43.Did you have a bloated feeling in your abdomen?	**1**	**2**	**3**	**4**
44.Did you have hot flushes?	**1**	**2**	**3**	**4**
45.Have you had sore or enlarged nipples or breasts?	**1**	**2**	**3**	**4**
46. Have you had swelling in your legs or ankles?	**1**	**2**	**3**	**4**

Please go to the next page

During the last 4 weeks…

	Not at all	A little	Quite a bit	Very much
47. Has weight **loss** been a problem for you?	1	2	3	4
48. Has weight **gain** been a problem for you?	1	2	3	4
49. Have you felt less masculine as a result of your illness or treatment?	1	2	3	4
50.To what extent were you interested in sex?	1	2	3	4
51. To what extent were you sexually active (with or without intercourse)?	1	2	3	4

PLEASE ANSWER THE NEXT FOUR QUESTIONS ONLY IF YOU HAVE BEEN SEXUALLY ACTIVE OVER THE LAST 4 WEEKS

52. To what extent was sex enjoyable for you?	1	2	3	4
53. Did you have difficulty getting or maintaining an erection?	1	2	3	4
54. Did you have ejaculation problems (eg dry ejaculation)?	1	2	3	4
55. Have you felt uncomfortable about being sexually intimate?	1	2	3	4

© Copyright 1999 EORTC Study Group on Quality of Life. All rights reserved. (phase III module)

Patient initials: ☐☐☐ Trial Number (*if applicable*): ☐☐☐

Date of Birth: ☐☐☐☐☐☐ Mrn: ☐☐☐☐☐☐☐☐☐

PROSTATE CANCER INSTITUTE

DIVISION OF CANCER SERVICES ST GEORGE HOSPITAL

ST. GEORGE HOSPITAL CANCER CARE CENTRE

Today Date: ____/____/_____

International Prostate Symptom Score (IPSS)
Instructions - Circle the most appropriate answer for each question

	Not at all	Less than 1 time in 5	Less than half the time	About half the time	More than half the time	Almost always
**1. Incomplete Emptying** Over the last month, how often have you had a sensation of not emptying your bladder completely after you finish urinating?	**0**	**1**	**2**	**3**	**4**	**5**
**2. Frequency** Over the last month, how often have you had to urinate again less than 2 hours after you finished urinating?	**0**	**1**	**2**	**3**	**4**	**5**
**3. Intermittency** Over the last month, how often have you found you stopped and started again several times when you urinated?	**0**	**1**	**2**	**3**	**4**	**5**
**4. Urgency** Over the last month, how often have you found it difficult to postpone urination?	**0**	**1**	**2**	**3**	**4**	**5**
**5. Weak Stream** Over the last month, how often have you had a weak urinary stream?	**0**	**1**	**2**	**3**	**4**	**5**
**6. Straining** Over the last month, how often have you had to strain or push to begin urination?	**0** None	**1** Once	**2** 2 times	**3** 3 times	**4** 4 times	**5** 5 or more times
**7. Nocturia** Over the last month, how many times did you most typically get up to urinate from the time you went to bed at night, until the time you got up in the morning?	**0**	**1**	**2**	**3**	**4**	**5**

***Total IPSS Score* =** ☐☐

	Delighted	Pleased	Mostly satisfied	Mixed	Mostly dissatisfied	Unhappy	Terrible
**8. Quality of Life due to Urinary Symptoms** If you were to spend the rest of your life with your urinary condition just the way it is now, how would you feel about that?	**0**	**1**	**2**	**3**	**4**	**5**	**6**

Please return this form to Dr Joseph Bucci, Cancer Care Centre, St George Hospital, Gray Street, Kogarah NSW 2217

Patient initials: ☐☐☐ Trial Number (*if applicable*): ☐☐☐

Date of Birth: ☐☐☐☐☐☐ Mrn: ☐☐☐☐☐☐☐☐☐

PROSTATE CANCER INSTITUTE

ST. GEORGE HOSPITAL CANCER CARE CENTRE

Today Date: ____/____/_____

International Index of Erectile Function (IIEF)

Instructions- Circle the most appropriate answer for each question

**1. How do you rate your <u>confidence</u> that you could get and keep and erection?**		Very Low<br>**1**	Low<br>**2**	Moderate<br>**3**	High<br>**4**	Very High<br>**5**
**2. When you had erections with sexual stimulation, <u>how often</u> were your erections hard enough for penetration (entering your partner) ?**	No sexual activity<br>**0**	Almost never or never<br>**1**	A few times (much less than ½ the time)<br>**2**	Sometimes (about ½ the time)<br>**3**	Most times (much more than ½ the time)<br>**4**	Almost always or always<br>**5**
**3. During sexual intercourse, <u>how often</u> were you able to maintain your erection after you had penetrated (entered) your partner?**	Did not attempt intercourse<br>**0**	Almost never or never<br>**1**	A few times (much less than ½ the time)<br>**2**	Sometimes (about ½ the time)<br>**3**	Most times (much more than ½ the time)<br>**4**	Almost always or always<br>**5**
**4. During sexual intercourse, <u>how difficult</u> was it to maintain your erection to completion of intercourse?**	Did not attempt intercourse<br>**0**	Extremely difficult<br>**1**	Very difficult<br>**2**	Difficult<br>**3**	Slightly difficult<br>**4**	Not difficult<br>**5**
**5. When you attempted sexual intercourse, <u>how often</u> was it satisfactory to you?**	Did not attempt intercourse<br>**0**	Almost never or never<br>**1**	A few times (much less than ½ the time)<br>**2**	Sometimes (about ½ the time)<br>**3**	Most times (much more than ½the time)<br>**4**	Almost always or always<br>**5**

IIEF Score = ☐☐

Please return this form to Dr Joseph Bucci, Cancer Care Centre, St George Hospital, Gray Street, Kogarah NSW 2217

Patient initials: ☐☐☐	Trial Number (*if applicable*): ☐☐☐
Date of Birth: ☐☐☐☐☐☐	Mrn: ☐☐☐☐☐☐☐☐☐

PROSTATE CANCER INSTITUTE

DIVISION OF CANCER SERVICES
ST GEORGE HOSPITAL

EPIC- Expanded Prostate Cancer Index Composite
Bowel Assessment

Today's date ____/____/_____

This questionnaire is designed to measure Quality of Life issues in patients with Prostate cancer. To help us get the most accurate measurement, it is important that you answer all questions honestly and completely.
Remember, as with all medical records, information contained within this survey will remain **strictly confidential**.

BOWEL HABITS- The next section is about your bowel habits and abdominal pain.
Please consider **ONLY THE LAST 4 WEEKS**.

1. How often have you had rectal urgency (felt like I had to pass stool, but did not) **during the last 4 weeks**? (Circle one number)

More than once a day	**1**
About once a day	**2**
More than once a week	**3**
About once a week	**4**
Rarely or never	**5**

2. How often have you had uncontrolled leakage of stool or feces? (Circle one number)

More than once a day	**1**
About once a day	**2**
More than once a week	**3**
About once a week	**4**
Rarely or never	**5**

3. How often have you had stools (bowel movements) that were loose or liquid (no form, watery, mushy) **during the last 4 weeks**? (Circle one number)

Never	**1**
Rarely	**2**
About half the time	**3**
Usually	**4**
Always	**5**

4. How often have you had bloody stools **during the last 4 weeks**? (Circle one number)

Never	**1**
Rarely	**2**
About half the time	**3**
Usually	**4**
Always	**5**

5. How often have your bowel movements been painful **during the last 4 weeks**? (Circle one number)

Never	**1**
Rarely	**2**
About half the time	**3**
Usually	**4**
Always	**5**

6. How many bowel movements have you had on a typical day **during the last 4 weeks**? (Circle one number)

Two or less	**1**
Three to four	**2**
Five or more	**3**

7. How often have you had crampy pain in your abdomen, pelvis or rectum **during the last 4 weeks**? (Circle one number)

More than once a day	**1**
About once a day	**2**
More than once a week	**3**
About once a week	**4**
Rarely or never	**5**

8. How big a problem, if any, has each of the following been for you? (Circle one number on each line)

	No Problem	Very Small Problem	Small Problem	Moderate Problem	Big Problem
a. Urgency to have a bowel movement	**0**	**1**	**2**	**3**	**4**
b. Increased frequency of bowel movements	**0**	**1**	**2**	**3**	**4**
c. Watery bowel movements	**0**	**1**	**2**	**3**	**4**
d. Losing control of your stools	**0**	**1**	**2**	**3**	**4**
e. Bloody stools	**0**	**1**	**2**	**3**	**4**
f. Abdominal/ Pelvic/Rectal pain	**0**	**1**	**2**	**3**	**4**

9. Overall, how big a problem have your bowel habits been for you **during the last 4 weeks**? (Circle one number)

No problem	**1**
Very small problem	**2**
Small problem	**3**
Moderate problem	**4**
Big problem	**5**
